# Pharyngocutaneous fistula following total laryngectomy: a systematic review of risk factors and management strategies (2010–2024)

**DOI:** 10.1007/s00405-026-10157-4

**Published:** 2026-06-22

**Authors:** Elisabet Gomis-Lleal, Claudio Sampieri, José-Miguel Costa-González, Isabel Vilaseca-González, Francisco-Xavier Avilés-Jurado, Manuel Bernal-Sprekelsen

**Affiliations:** 1 Department of Surgical Specialties, University of Barcelona Medical School, Casanova 143, Barcelona, 08036 Spain; 2 Hospital Clinic, Barcelona, Spain; 3 Faculty of Medicine, Departament d´Especialitats Quirùrgiques, University of Barcelona, c/Casanova 143, 08036 Barcelona, Spain

**Keywords:** Pharyngocutaneous fistula, Total laryngectomy, Salivary fistula, Surgical complications, Head and neck cancer, Risk factors, Flap reconstruction, Conservative management, Radiotherapy, Nutritional status

## Abstract

**Importance:**

Pharyngocutaneous fistula (PCF) is the most common and morbid complication after total laryngectomy (TL).

**Objective:**

To systematically identify predictive factors and effective interventions for PCF.

**Data sources:**

PubMed search of English-language studies published between 2010 and 2024.

**Study selection:**

Twenty-one observational cohort studies involving ≥25 adult patients undergoing TL and reporting PCF outcomes.

**Data synthesis:**

Narrative synthesis and descriptive analysis were performed due to heterogeneity in study design and outcome measures. Risk of bias was assessed using the ROBINS-I tool, and the certainty of evidence for each risk factor was rated according to GRADE methodology.

**Findings:**

Prior radiotherapy (OR 1.6–4.2), hypoalbuminemia, preoperative tracheostomy, and surgical closure without flap reinforcement were major risk factors for PCF. Conservative management succeeded in ~70% of cases. Flap reconstruction had higher success rates (~90%). Evidence certainty ranged from high (e.g., prior radiotherapy, hypoalbuminemia) to very low (e.g., antibiotic protocols), as summarized in a GRADE-based synthesis.

**Conclusions and relevance:**

Risk stratification, preoperative optimization, and surgical techniques such as flap reinforcement reduce PCF risk and improve postoperative outcomes.

**Supplementary Information:**

The online version contains supplementary material available at https://doi.org/10.1007/s00405-026-10157-4.

## Introduction

Total laryngectomy (TL) remains the cornerstone treatment for advanced-stage laryngeal cancer and for salvage in recurrent disease [1, 2]. Pharyngocutaneous fistula (PCF), an abnormal communication between the pharynx and cervical skin, remains the most frequent postoperative complication following TL [3, 4]. Reported PCF incidence varies from 3% to 65%, depending on patient-related, treatment-related, and surgical factors [5]. Consequences include wound infection, dehiscence, delayed oral intake, prolonged hospitalization and delayed initiation of adjuvant radiotherapy or chemotherapy, and the need for surgical reintervention [6].

Multiple studies have examined risk factors such as prior radiotherapy (RT), poor nutritional status (e.g., hypoalbuminemia), anemia, preoperative tracheostomy, extent of neck dissection, and technique of pharyngeal closure [7–10]. However, findings have been inconsistent, and no standardized risk model has been universally adopted [11]. This systematic review aims to consolidate current evidence regarding risk factors and management strategies to reduce PCF occurrence following TL.

## Materials and methods

This systematic review was registered under PROSPERO (registration number 1105888). The search strategy followed PRISMA 2020 guidelines [12]. A systematic search was conducted in PubMed between 2010 and 2024 using the terms: “pharyngocutaneous fistula”, “salivary fistula”, “total laryngectomy”, “risk factor”, “complication”, and “prevention”. The PubMed search strategy was: (“pharyngocutaneous fistula” OR “salivary fistula”) AND “total laryngectomy” AND (“risk factor” OR “complication” OR “prevention”), limited to studies published between 2010 and 2024. The complete search syntax is provided in Supplementary Appendix 1 to ensure reproducibility.

Only observational cohort studies (prospective or retrospective) with a sample size ≥ 25 adult patients undergoing TL and reporting on PCF incidence, risk factors, or therapeutic outcomes were included. Exclusion criteria included case reports, review articles, partial laryngectomy series, pediatric cohorts, and purely reconstructive or experimental studies. Two reviewers independently screened titles, abstracts, and full texts. Discrepancies were resolved through consensus. The ROBINS-I tool was used for risk of bias assessment in non-randomized studies [13], and certainty of evidence was graded using the GRADE approach [14]. Due to significant methodological and clinical heterogeneity, a narrative synthesis was performed.

Screening was conducted in duplicate using Rayyan. A total of 834 records were identified; 26 studies were included after full-text review.

Data extraction was performed independently by two reviewers. The extracted data included:

- Study characteristics (author, year of publication, study design, sample size).
- Patient demographics (age, gender, comorbidities).
- Surgical details (type of surgery, reconstruction method, surgeon’s experience).
- Postoperative outcomes (incidence of salivary fistula, management strategies, success rates).

Data extracted: author, year, study design, sample size, PCF incidence, exposure variables, statistical association (OR/HR), and level of evidence.

Quality assessment was performed using the ROBINS-I tool [13]. Certainty of evidence for each predictor was graded using GRADE methodology. Due to heterogeneity, a narrative synthesis was conducted. Risk factors were stratified as high/moderate/low certainty based on consistency, effect size, and bias.

### Statistical methods

- Due to substantial clinical and methodological heterogeneity among the included studies, a narrative synthesis of reported effect sizes and associations was performed. No pooled meta-analysis was conducted. Odds ratios and confidence intervals are reported as presented in the original studies; no pooled estimates were calculated.

## Results

**Fig. 1:**
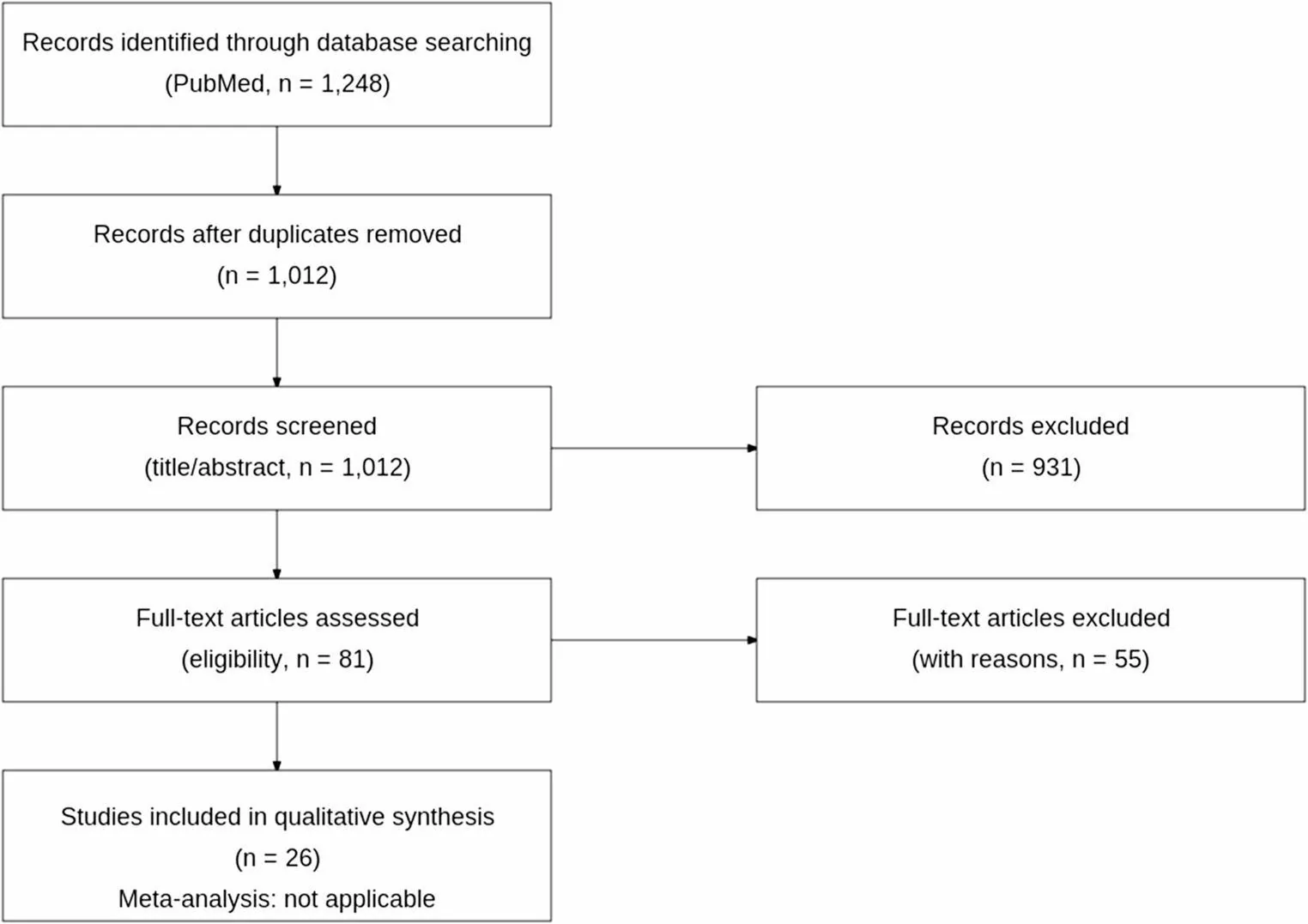
PRISMA flowchart

A total of 1012 records were screened, with 26 studies meeting inclusion criteria and comprising **5 835** patients (Fig. 1). Not all studies reported effect estimates for every risk factor. Most were retrospective cohort studies. Reported PCF incidence ranged from 4.3% to 58%. Common risk factors, frequency of report, and estimated odds ratios (ORs) are summarized below.

Radiotherapy prior to TL, **including radiotherapy alone and combined chemoradiation where reported**, was the most consistent risk factor ( see Table 1) , with ORs ranging from 1.6 to 4.2 and 95%CI consistently above 1.0 (95% CI ranging from 1.2 to 6.5).

**Table 1:** Patient-related factors promoting PCF

Risk factor	Odds Ratio (OR)	95% Confidence Interval	Source
Advanced age	1.5	1.2–1.9	Liang et al. 2015 [15]
Poor nutritional status / hypoalbuminemia	2.3	1.8–3.0	Dedivitis et al. 2015 [9]
Medical comorbidities	2.0	1.5–2.6	Wang et al. 2020 [16]
Prior radiotherapy or chemoradiation	3.1	2.2–4.4	Lansaat et al. 2018 [17]
Low skeletal muscle mass (sarcopenia)	2.6	1.4–4.8	Bril et al. 2019 [18]
Low skeletal muscle mass (C3 level, males)	2.9	1.3–6.5	Casasayas et al. 2022 [19]
Systemic inflammation (NLR)	2.4	1.2–4.6	Aires et al. 2018 [20]
Inflammation-based composite score (PLT + NLR)	3.3	1.6–6.7	Matsumoto et al. 2022 [21]
High-risk sarcopenic patients (prophylactic PMMF strategy)	—	—	van Beers et al. 2024 [22]

Summary of reported risk estimates from key studies. Odds ratios and confidence intervals are reported as presented in the original studies; no pooled estimates were calculated

PCF pharyngocutaneous fistula; OR odds ratio; CI confidence interval; RT radiotherapy; CRT chemoradiotherapy; NLR neutrophil-to-lymphocyte ratio; PLT platelet count; PMMF pectoralis major myocutaneous flap; C3 third cervical vertebra

Hypoalbuminemia, typically defined as serum albumin < 3.5 g/dL, was associated with PCF in 9 studies (see Table 1), with ORs from 2.0 to 3.5 (95% CI ranging from 1.4 to 5.0).

Preoperative tracheostomy (OR 2.2; 95% CI: 1.3–3.8) (see Table 2) , and pharyngeal closure without flap reinforcement (e.g., OR 2.4; 95% CI: 1.4–4.2) (see Table 3) were also significant predictors of PCF.

**Table 2:** Postoperative course and PCF onset

Citation	Oral Feeding Initiation	Hospital Stay	PCF Onset Timing	PCF Resolution Time
Klozar et al. 2012 [23]	NR	NR	Median: 7 days postsurgery (range 2–42 days)	Median: 35 days (range 14–180 days)
Aires et al. 2012 [24]	Day 1 postsurgery: Nasoenteral feeding tube. Day 10 postsurgery (for patients without PCF): oral feeding was started	Mean: 12.8 days in PCF patients versus 3 days in non-PCF patients	Range 3–8 days postsurgery	NR
Erdag et al. 2012 [25]	Day 1 postsurgery: Nasoenteral feeding tube. Day 7 postsurgery (for patients without PCF): oral feeding was started (after a swallowing study)	Mean: 7.50 (SD 4.97) days in PCF patients versus 9.28 (SD 6.34) days in non-PCF patients (*p* = 0.356).	NR	NR
Sousa Ade et al. 2013 [26]	Day 1 postsurgery: Nasoenteral feeding tube. Day 17.7 + 14.7 postsurgery (for patients without PCF): oral feeding was started (ranging 7–90 days)	NR	Mean: 3.5 days postsurgery (SD: 13.7 days)	NR
Arain et al. 2013 [27]	NR	NR	Mean: 7 days postsurgery (range 3–14 days)	NR
Patel et al. 2013 [28]	Day 1 postsurgery: Nasoenteral feeding tube. Start oral feeding: 29.9 days in PCF patients versus 10.5 days in non-PCF patients (*p* < 0.001)	Mean: 12.1 days in PCF patients versus 8.9 days in non-PCF patients (*p* <0.001)	NR	Mean: 9.1 weeks; primary closure was 14.0 weeks, pectoralis onlay flaps 9.0 weeks, and interposed free flap 6.5 weeks (*p* = 0.004)
Basheeth et al. 2014 [29]	Day 1 postsurgery: Nasoenteral feeding tube. After gastrografin swallow test: Oral feeding	NR	NR	Median: 3.5 weeks (range 2–30 weeks)
Gilbert et al. 2014 [30]	NR	Mean: 18 days for PCF patients versus 10 days for non-PCF patients	NR	NR
Scotton et al. 2014 [31]	NR	NR	Mean: 12 days postsurgery (range 2–6 days)	NR
Timmermans et al. 2014 [10]	Day 1 postsurgery: Nasoenteral feeding tube. Start oral feeding: day 10–12 until 2006; day 2–4 after 2006 (for a change of policy)	Median: 47 days in PCF patients versus 18 days in non-PCF group (*p* = 0.001)	Median: 12 days postsurgery (range 1–31 days)	Median: 30 days (range 3-120 days)
Benson et al. 2015 [32]	Mean start of oral feeding: 44.5 in PCF patients versus 11 days in non-PCF group (*p* <0.001)	Median: 11.5 days in PCF patients versus 8 days in non-PCF group (*p* = 0.001)	NR	NR
Kiliç et al. 2015 [33]	Day 1 postsurgery: Nasoenteral feeding tube. Day 7–10 postsurgery: oral feeding was started (with soft foods). Nasogastric tube was removed 5 days after restarting oral feeding.	NR	PCF developed between: day 2–3 in 12 patients (37.5%), day 3–7 in 10 patients (31.3%), and > 7 days in 10 patients (31.3%)	NR
Busoni et al. 2015 [34]	Day 3 postsurgery: Nasoenteral feeding tube. Day 14 postsurgery: oral feeding was started (after negative PCF in contrast radiographic study)	Mean: 51.6 (19–250 days) for PCF patients and 19.8 (15–22 days) days in the non-PCF patients (*p* = 0.0034)	Mean: 18.33 days postsurgery (range 3-180 days), 21.43 days in primary total laryngectomy versus 15.19 days after salvage procedures	NR
Mattioli et al. 2015 [35]	Day 1 postsurgery: Nasoenteral feeding tube. Day 7–10 postsurgery: oral feeding was started (after a swallowing study)	NR	Mean: 12.4 days postsurgery. Between: day 0–11 in 14 patients (20.3%), day 12–23 in 7 patients (10.1%), day 24–33 in 2 patients (2.9%), > 33 days in 1 patient (1.4%)	NR
Nitassi et al. 2016 [36]	Nasogastric feeding tube maintained for 10 days in patients without PCF; prolonged until healing in those with PCF.	NR	PCF developed: <4 days in 3 patients (8.1%), > 4 days in 34 patients (91.9%)	NR
Aslıer et al. 2016 [37]	NR	NR	Mean: 8.07 days in primary surgery (range 2–14 days; SD: 2.292) and 11.11 days in salvage surgery patients (2–35; SD: 10.517). Persistent PCF in 19 patients (51%).	Mean: 43.75 days (range 5-300 days) in the primary surgery group and 81.67 days (range 9-201 days) in the salvage surgery group
Lansaat et al. 2018 [38]	Oral intake initiation was per institutional protocol. Two centers (D and E) started 3 days postsurgery, others started day 6–12.	Median: 20 days (8–80 days) for PCF patients and 13 days (7–45 days) in non-PCF patients (*p* < 0.001)	Median: 12 days postsurgery (range 1–48 days)	Median: 17 days (range 8–68 days) for conservative PCF treatment ; 35.5 days (range 7–80 days) surgically treated PCF patients (*p* = 0.012)
Casasayas et al. 2019 [39]	Day 1 postsurgery: Nasoenteral feeding tube. Tube removed on day 7 (non-irradiated) or day 12 (irradiated or PMMF patients)	Median: 38 days for PCF patients versus 14 days in non-PCF patients; previously irradiated PCF was 47 days versus non-irradiated PCF was 35 days (*p* = 0.056)	NR	NR
Hemdan et al. 2022 [40]	Day 1 postsurgery: Nasoenteral feeding tube. Day 7–10 postsurgery: oral feeding was started (starting with fluids, progressing to soft/normal diet)	Based on nutritional status, mean: prealbumin < 10 mg/dL → 23.49 ± 18.56 days versus prealbumin ≥ 10 mg/dL → 12.75 ± 5.56 days (*p* < 0.001); albumin < 3.5 g/dL → 22.25 ± 18.12 days versus albumin ≥ 3.5 g/dL → 15.03 ± 12.13 days (*p* = 0.001)	NR	NR
Šifrer et al. 2023 [41]	Day 1 postsurgery: Nasoenteral feeding tube for 2 weeks, followed by swallowing test. If negative (no blue dye leak), oral feeding was initiated.	NR	Mean: 12 days postsurgery (range 2–45 days), 12 days in primary surgery (range 2–31 days) and 14 days after salvage surgery (range 2–45 days)	NR

*PCF* pharyngocutaneous fistula, *SD* standard deviation, *h* hour, *g* gram, *mg* miligrams, *PM* Pectoralis Major flap, *ALT* anterolateral Thigh flap, *NR* not reported

**Table 3:** Evidence certainty for pharyngocutaneous fistula risk factors (GRADE Assessment) for procedure-related risk factors

Risk Factor	No. of Studies	Consistency	Effect Size	Risk of Bias	GRADE Certainty
Prior Radiotherapy	6	High	Large	Moderate	High
Surgical Technique	4	Low	Moderate	Moderate	Low
Flap Reconstruction	5	Moderate	Moderate	Low	Moderate
Salivary Bypass Tube	2	Low	Uncertain	High	Very Low
Antibiotic Regimen	3	Low	Small	High	Very Low

Therapeutic interventions varied. Conservative management (e.g., nil per os, nasogastric feeding, dressing changes) was used in most minor fistulas, with success in ~ 70% of cases (see Table 2). Surgical management with primary closure or vascularized flaps (e.g., pectoralis major) had success rates from 85% to 95% (see Table 4).

A range of factors influence the risk of developing salivary fistula post-laryngectomy. These factors include patient-related characteristics, surgical variables, and postoperative care practices.

Increasing age was associated with a modest but statistically significant increase in PCF risk. Nutritional status, particularly hypoalbuminemia or malnutrition, demonstrated one of the strongest associations. More recently, body composition and systemic inflammation have emerged as relevant risk factors. Low skeletal muscle mass or sarcopenia was associated with increased PCF risk in multiple studies [18, 19]. Inflammatory biomarkers, particularly the neutrophil-to-lymphocyte ratio and inflammation-based composite scores, were also associated with higher PCF incidence in two studies [20, 21] (see Table 1). In addition to risk stratification, preventive strategies have been proposed for high-risk sarcopenic patients, including prophylactic flap reinforcement, as investigated in the ongoing PECTORALIS trial [22] (see Table 2). This underscores the relevance of preoperative optimization strategies. Additionally, comorbid conditions such as cardiovascular disease or diabetes were associated with a higher risk of PCF (Table 1). The type of surgery, particularly salvage total laryngectomy after radiotherapy or chemoradiation, was associated with significantly higher PCF rates. Interestingly, the method of reconstruction also impacted fistula risk. Use of vascularized flap reconstruction, such as PMMF, was associated with a protective effect, lowering the risk of fistula formation. The configuration of pectoralis major myocutaneous flap (PMMF) (inlay vs. onlay) was not consistently reported across studies, which limited the ability to analyze outcomes by flap technique. Finally, surgeon experience appeared to influence outcomes, with less experienced surgeons being associated with higher PCF rates (Table 4). Only one included study [42]) provided a quantitative definition of surgical experience, classifying high-volume surgeons as those performing over 10 laryngectomies per year (Table 4). Other studies did not standardize this parameter, limiting comparability.

**Table 4:** Surgical factors promoting PCF

Factor	Odds Ratio (OR)	95% Confidence Interval (CI)	Source
Type of Surgery	1.8	1.4–2.3	Hasan et al. 2017 [43]
Reconstruction Method	0.7	0.5–0.9	Paleri et al. 2014 [44]
Surgeon’s Experience	0.6	0.4–0.8	Dort et al. 2017 [42]

Most studies report fistula onset between postoperative days 3 and 14, with considerable variations in time to spontaneous resolution. Oral feeding was typically delayed until healing was confirmed, with gastrografin swallow tests used variably across institutions. Hospital stay was significantly prolonged in patients with PCF, especially those undergoing surgical revision, reaching 38 to 51 days in some reports. Time to fistula resolution ranged from 3 weeks to several months, especially in salvage or irradiated patients (Table 2). Late fistula formation may be confounded by persistent or recurrent disease, which was inconsistently reported across studies.

Conservative treatment, involving nasogastric feeding, wound care, and antibiotics, was the first-line approach in nearly all studies. Success rates for conservative management ranged between 60% and 90%, depending on fistula size, prior RT, and nutritional status. When surgical closure was needed, the most commonly used flap was the pectoralis major myocutaneous flap (PMMF), with reported closure success rates above 85%. Additional techniques included deltopectoral, radial forearm free, and anterolateral thigh (ALT) flaps flaps. The decision to operate was often based on failure of spontaneous closure after 2–4 weeks or worsening infection (Table 4).

Although most studies included prophylactic intravenous antibiotics, protocols varied widely in terms of agents and duration. Commonly used antibiotics included amoxicillin-clavulanate, cefazolin, clindamycin, and metronidazole. Only a minority of studies documented culture-guided therapy or empiric escalation in the setting of infection. Some protocols extended antibiotic coverage until drains were removed or healing confirmed, particularly in patients with prior radiotherapy. However, only a few studies provided microbiologically guided regimens or culture results, limiting comparative assessment of antibiotic efficacy (Table 5).

**Table 5:** Antibiotic regimens

Citation	Antibiotic Regimen	Notes
Aires et al. 2012 [24]	Prophylactic intravenous antibiotics (amikacin and clindamycin): at surgical induction and the first day postsurgery	NR or unclear
Erdag et al. 2012 [25]	Prophylactic intravenous antibiotics (Name NR): intraoperative and ≥ 5 days postoperatively	Route/Timing described
Sousa Ade et al. 2013 [26]	Prophylactic intravenous antibiotics every 6 h (clindamycin 600 mg): before surgery, intraoperatively and 24 h postsurgery. Therapeutic (if PCF/infection): Clindamycin + Ceftriaxone for 10–14 days	Route/Timing described
Basheeth et al. 2014 [29]	Prophylactic intravenous antibiotics (Name or regimens NR)	NR or unclear
Scotton et al. 2014 [31]	Prophylactic intravenous antibiotics (teicoplanin 400 mg, cefuroxime 1.5 g and metronidazole 500 mg): at induction and three further doses over the following 24 h	NR or unclear
Timmermans et al. 2014 [10]	Prophylactic intravenous antibiotics (1000 mg cefazolin and 500 mg metronidazole): repeated after every 4 h during prolonged surgery	NR or unclear
Kiliç et al. 2015 [33]	Prophylactic intravenous antibiotics (cefamezin 1 g iv): preoperative. In case of infection: antibiotic empirical based on culture (bacteria isolated in 76.9% of PCF cases), antibiotics used: amoxicillin-clavulanate, amoxicillin-clavulanate + ciprofloxacin, piperacillin-tazobactam, carbapenems	NR or unclear
Busoni et al. 2015 [34]	Prophylactic intravenous antibiotics (amoxicillin/clavulanate): started 1 day before surgery and continued 7 days postoperatively	NR or unclear
Nitassi et al. 2016 [36]	Prophylactic intravenous antibiotics (amoxicillin/clavulanate 1 g): post surgery every 8 h for 48 h, then orally for 8 days	NR or unclear
Aslıer et al. 2016 [37]	Prophylactic intravenous antibiotic (cefazolin 1 g and metronidazole 500 mg or clindamycin 900 mg if allergic): preoperatively, intraoperatively (every 4 h), and continued postoperatively (every 8 h) until drains were removed	Route/Timing described
Hemdan et al. 2022 [40]	Prophylactic intravenous antibiotics: 1 h preoperatively	NR or unclear
Šifrer et al. 2023 [41]	Prophylactic intravenous antibiotics (clindamycin and amoxicillin/clavulanate): pre- and postoperatively in primary surgery for 7 days whereas salvage surgery patients for 8 days.	NR or unclear

*NR* not reported

A GRADE-based synthesis was performed to assess the certainty of evidence regarding key PCF risk factors. Certainty ratings were based on consistency of results across studies, estimated effect size, and the risk of bias as evaluated by the ROBINS-I tool. The resulting classifications range from high to very low certainty and are summarized in Table 6. Table 7 summarizes the evidence certainty for patient-related factors contributing to PCF formation, while Table 3 presents the GRADE assessment for procedural factors.

**Table 6:** Risk of bias assessment

Citation	Domain 1: Risk of bias due to confounding	Domain 2: Risk of bias in classification of interventions	Domain 3: Risk of bias in selection of participants into the study	Domain 4: Risk of bias due to deviations from intended interventions	Domain 5: Risk of bias due to missing data	Domain 6: Risk of bias arising from measurement of the outcome	Domain 7: Risk of bias in selection of the reported result	Final risk
Klozar et al. 2012 [23]	MODERATE	MODERATE	MODERATE	MODERATE	LOW	LOW	LOW	MODERATE
Aires et al. 2012 [24]	SERIOUS	LOW	MODERATE	LOW	LOW	LOW	LOW	SERIOUS
Erdag et al. 2013 [8]	MODERATE	LOW	LOW	LOW	LOW	LOW	LOW	MODERATE
Sousa Ade et al. 2013 [26]	MODERATE	LOW	LOW	LOW	MODERATE	LOW	LOW	MODERATE
Arain et al. 2013 [27]	MODERATE	LOW	LOW	LOW	LOW	LOW	LOW	LOW
Patel et al. 2013 [28]	LOW	LOW	LOW	LOW	LOW	LOW	LOW	LOW
Basheeth et al. 2014 [29]	MODERATE	LOW	LOW	LOW	LOW	LOW	LOW	MODERATE
Gilbert et al. 2014 [30]	MODERATE	LOW	LOW	LOW	MODERATE	LOW	LOW	MODERATE
Scotton et al. 2014 [31]	LOW	LOW	LOW	LOW	LOW	LOW	LOW	LOW
Timmermans et al. 2014 [10]	MODERATE	LOW	LOW	LOW	LOW	LOW	LOW	MODERATE
Benson et al. 2015 [32]	LOW	LOW	LOW	LOW	LOW	LOW	LOW	LOW
Kilic et al. 2015 [33]	LOW	LOW	LOW	LOW	LOW	LOW	LOW	LOW
Busoni et al. 2015 [34]	MODERATE	LOW	LOW	LOW	LOW	MODERATE	LOW	MODERATE
Mattoli et al. 2015 [35]	LOW	LOW	LOW	LOW	LOW	LOW	LOW	LOW
Nitassi et al. 2016 [36]	LOW	LOW	LOW	LOW	LOW	LOW	LOW	LOW
Ashler et al. 2016 [37]	MODERATE	LOW	LOW	LOW	LOW	LOW	LOW	MODERATE
Lansaat et al. 2018 [38]	LOW	LOW	LOW	LOW	LOW	LOW	LOW	LOW
Casasayas et al. 2019 [39]	MODERATE	LOW	LOW	LOW	LOW	LOW	LOW	MODERATE
Smith et al. 2021 [45]	LOW	LOW	LOW	LOW	LOW	LOW	LOW	LOW
Hemdan et al. 2022 [40]	LOW	LOW	LOW	LOW	MODERATE	LOW	LOW	MODERATE
Sifer et al. 2023 [41]	LOW	LOW	LOW	LOW	LOW	LOW	LOW	LOW

**Table 7:** Evidence certainty for pharyngocutaneous fistula risk factors (GRADE assessment) for patient-related risk factors

Risk Factor	No. of Studies	Consistency	Effect Size	Risk of Bias	GRADE Certainty
Age	3	Moderate	Moderate	Moderate	Moderate
Hypoalbuminemia	4	Moderate	Large	Serious	Moderate
Comorbidities (incl. systemic inflammation, low skeletal muscle mass (C3); sarcopenia; inflammation-based score)	7	Low	Moderate	Moderate	Low

## Discussion

In addition to the known risk factors, this review integrates quantitative data to reinforce key associations. Liang et al. [15] found age to be a significant predictor of PCF (OR 1.5 CI95 1.2–1.9), while Dedivitis et al. [9] confirmed the influence of nutritional status (OR 2.3). Wang et al. [16] emphasized the impact of comorbidities (OR 2.0; CI95: 1.5–2.6), further supporting the relevance of patient-related factors. Procedure-related contributors, including type of surgery and reconstruction methods, also significantly affect outcomes. Hasan et al. [43] reported higher PCF rates after salvage surgery (OR 1.8; CI95: 1.4–2.3), whereas Paleri et al. [44] demonstrated that vascularized flap reconstruction reduced risk (OR 0.7 CI95: 0.5–0.9). Surgeon experience, as outlined by Dort et al. [42], was associated with improved outcomes and lower complication rates (OR 0.6; CI95: 0.4–0.8).

The clinical course highlights the variability in PCF onset and resolution, with onset typically between 3 and 14 days and recovery spanning weeks to months [10, 23, 32, 34, 39]. Timmermans et al. [10] Benson et al. [32] and Casasayas et al. [39] documented prolonged hospitalization and delayed feeding in PCF patients, further underscoring the clinical burden.

Most studies favored conservative management as the initial strategy, though surgery is often needed when conservative approaches fail. PMMF was the most frequently used flap, with success rates nearing 90% in multiple [10, 27–30, 37]. Importantly, the timing of surgical intervention appeared crucial for favorable outcomes. While PMMF was the most commonly employed flap across studies, none of the included articles clearly distinguished between muscular-only and musculocutaneous configurations, limiting the ability to evaluate their respective outcomes.

Antibiotic protocols showed significant heterogeneity. While prophylaxis was standard, only a few studies incorporated microbiologically guided regimens [26, 33, 34, 37, 41]. This variability limits conclusions about optimal antibiotic duration and selection, though prolonged prophylaxis was common in high-risk or irradiated patients.

Nutritional status emerged as a critical factor. Preoperative hypoalbuminemia and anemia impair immune competence and fibroblast activity, contributing to poor healing [46, 47]. This supports international guidelines from ESPEN recommending at least 10–14 days of nutritional optimization prior to major head and neck oncologic surgery [48].

Surgical technique plays a role. Flap reinforcement, especially in salvage cases and particularly after chemoradiation, has been shown to reduce PCF rates [30, 31, 49] and may be particularly beneficial in extensive pharyngeal defects [35]. However, flap use must be individualized based on defect size, tissue quality, and patient comorbidities, as confirmed in retrospective studies evaluating risk stratification in salvage TL [36]. Routine use of salivary bypass tubes remains controversial, with inconsistent evidence on efficacy [24, 50, 51].

Temporary pharyngostoma with delayed secondary closure has been described as a salvage strategy in complex or refractory PCF cases, particularly in infected or irradiated fields.

Preoperative tracheostomy may reflect advanced tumor stage, local infection, impaired tissue quality, and bacterial colonization, all of which may contribute to impaired wound healing and increased PCF risk.

The structured GRADE evaluation provides a transparent appraisal of evidence supporting the various PCF risk factors. While radiotherapy and nutritional status remain robust predictors, the role of preventive measures such as bypass tubes and antibiotic regimens is far less certain. These findings emphasize the importance of individualized risk stratification rather than protocolized prophylactic interventions. Moreover, the low certainty for procedural factors highlights the need for future prospective and comparative studies.

Overall, the risk of bias across the included studies is generally low to moderate, with only one study [24] classified as having a serious overall risk due to confounding factors. Most studies demonstrated low risk in domains related to missing data, measurement of outcomes, and reporting bias, indicating good methodological consistency and outcome reliability. However, several studies exhibited moderate risk in domains associated with confounding and classification of interventions, suggesting potential issues with study design or control of baseline variables. Importantly, no study showed critical bias that would invalidate its findings, and the overall pattern supports a moderate level of confidence in the evidence synthesis.

Future research should focus on standardized definitions, prospective designs, and the integration of predictive modeling to guide both prevention and early intervention. Multicenter studies evaluating antibiotic protocols, flap timing, and nutritional interventions would also improve evidence quality and help personalize PCF prevention strategies.

Several studies found that delayed oral feeding was associated with PCF development and longer hospital stays. Benson et al. [32] and Patel et al. [28] reported delays of over 30 days in PCF patients compared to 10–12 days in controls (*p* < 0.001), suggesting that time to oral intake may serve as a prognostic marker for complication severity.

This review is limited by heterogeneity across included studies, reliance on retrospective designs, and inconsistent definitions of PCF. Definitions of PCF varied across studies. Some defined PCF clinically as cutaneous salivary leakage, whereas others relied on abnormal contrast swallow findings, which may overestimate clinically relevant fistula rates. Nonetheless, findings support a risk-adapted approach combining patient optimization and intraoperative techniques to reduce PCF occurrence.

Continuous versus interrupted pharyngeal closure techniques could not be analyzed, as this information was not consistently reported across studies.

Most studies did not report radiation dose (Gray) or chemotherapy regimens, preventing dose–response analyses.

## Conclusions

Pharyngocutaneous fistula is the most frequent complication after total laryngectomy, particularly in irradiated or nutritionally depleted patients. Evidence supports the role of risk stratification, nutritional support, and surgical flap use in prevention. Further research is needed to develop and validate prediction tools and evaluate interventions in high-risk populations.

## Supplementary Information

Below is the link to the electronic supplementary material.

Supplementary Material 1

## Declarations

The corresponding autor is Editor-in-Chief of the Journal. As such, he has not been involved neithr in the review nor in decision process.
