# Supplementary material for: Pharyngocutaneous fistula following total laryngectomy: a systematic review of risk factors and management strategies (2010–2024)
Source: Eur Arch Otorhinolaryngol. 2026 Jun 22;283(7):4609–18. doi: 10.1007/s00405-026-10157-4 (PMC13388526; doi:10.1007/s00405-026-10157-4)
Supplement: Supplementary file 1 — Supplementary Material 1 [file 405_2026_10157_MOESM1_ESM.docx]

**Supplementary Appendix 1**

**Detailed Literature Search Strategy**

A comprehensive and systematic literature search was performed to identify studies evaluating risk factors, prevention strategies, and outcomes related to pharyngocutaneous fistula following total laryngectomy.

**Databases searched**

The primary electronic database searched was **PubMed/MEDLINE**. Reference lists of included articles and relevant reviews were also manually screened to identify additional eligible studies.

**Time frame**

The search covered publications from **1 January 2010 to 31 December 2024**.

**Language restrictions**

Only articles published in **English** were considered eligible.

**Search strategy**

The PubMed search strategy was developed using a combination of Medical Subject Headings (MeSH) terms and free-text keywords related to pharyngocutaneous fistula and total laryngectomy. Boolean operators were used to combine search terms.

The full PubMed search string was as follows:

("pharyngocutaneous fistula"[Title/Abstract]

OR "pharyngo-cutaneous fistula"[Title/Abstract]

OR "salivary fistula"[Title/Abstract])

AND

("total laryngectomy"[Title/Abstract]

OR "laryngectomy"[Title/Abstract])

AND

("risk factor"[Title/Abstract]

OR "predictor"[Title/Abstract]

OR "complication"[Title/Abstract]

OR "outcome"[Title/Abstract]

OR "prevention"[Title/Abstract])

Filters applied: Publication dates from 2010/01/01 to 2024/12/31; Humans; English.

**Study selection**

All records identified through the search were imported into a reference management software and duplicates were removed. Titles and abstracts were screened independently by two reviewers. Full texts of potentially relevant studies were then assessed for eligibility.

**Eligibility criteria**

Studies were included if they met the following criteria:

- Original clinical studies (randomized trials, prospective or retrospective cohort studies, case–control studies)
- Adult patients undergoing total laryngectomy
- Reported pharyngocutaneous fistula as an outcome
- Evaluated at least one risk factor, preventive strategy, or management aspect related to pharyngocutaneous fistula

Exclusion criteria were:

- Case reports, small case series (<10 patients), editorials, letters, and conference abstracts
- Studies not reporting extractable data on pharyngocutaneous fistula
- Non-human studies

**Data extraction and synthesis**

Data extraction was performed independently by two reviewers. Due to substantial clinical and methodological heterogeneity across studies, a **narrative synthesis** was conducted. No pooled quantitative meta-analysis was performed.

**Risk of bias and certainty of evidence**

Risk of bias was assessed using the **ROBINS-I** tool for non-randomized studies. The certainty of evidence for each risk factor was evaluated using the **GRADE** approach and summarized in Tables 3 and 8.
